# Associations Between Average Activity Variety and Average Cognitive Performance Across 14 Days

**DOI:** 10.1093/geroni/igaf122.1605

**Published:** 2025-12-31

**Authors:** Allison Bielak, Jacqueline Mogle, Martin Sliwinski

**Affiliations:** Colorado State University, Fort Collins, Colorado, United States; RTI Health Solutions, Durham, North Carolina, United States; The Pennsylvania State University, University Park, Pennsylvania, United States

## Abstract

There is some evidence that greater diversity of activity across the day is positively associated with that day’s working memory performance. However, past work only permitted choosing a single, currently engaged in activity at each assessment, providing a limited index of activity variety. Using tablet-based assessments, 81 community-dwelling participants 41-94 years of age (M = 61.26, SD = 12.12) reported up to 20 activities they participated in the past 3-4 hours, and concurrently completed 3 cognitive tasks (2-back working memory, spatial working memory, processing speed) 4 times per day for 14 days. Multilevel models covarying age, gender, education, and retirement status showed that within-person fluctuations in average activity variety per session did not covary with average cognitive performance per session. Greater between-person average activity variety was associated with faster 2-back working memory on day 1, but those with higher average activity variety also showed less improvement over days for both working memory tasks. A detrimental association was also found for those in their 40s where greater average activity variety was associated with lower day 1 processing speed and spatial working memory. The limited associations with average activity variety and average cognitive performance were unexpected, along with variation in findings by cognitive outcome and age group. Associations with average activity variety appear to be more relevant than daily fluctuations in activity variety. Other work has showed short-term covariation between activity and cognition over the day when a frequency-based activity measure was used. Further inquiry into the type of activities engaged in may yield clearer results.

